# The synovial and blood monocyte DNA methylomes mirror prognosis, evolution, and treatment in early arthritis

**DOI:** 10.1172/jci.insight.158783

**Published:** 2022-05-09

**Authors:** Carlos de la Calle-Fabregat, Javier Rodríguez-Ubreva, Laura Ciudad, Julio Ramírez, Raquel Celis, Ana Belén Azuaga, Andrea Cuervo, Eduard Graell, Carolina Pérez-García, César Díaz-Torné, Georgina Salvador, José A. Gómez-Puerta, Isabel Haro, Raimon Sanmartí, Juan D. Cañete, Esteban Ballestar

**Affiliations:** 1 Epigenetics and Immune Disease Group, Josep Carreras Research Institute (IJC), Badalona, Barcelona, Spain.; 2 Epigenetics in Inflammatory and Metabolic Diseases Laboratory, Health Science Center (HSC), East China Normal University (ECNU), Shanghai, China.; 3 Arthritis Unit, Rheumatology Department, Hospital Clinic and IDIBAPS, Barcelona, Spain.; 4 Rheumatology Department, Hospital Universitari Parc Taulí, Sabadell, Barcelona, Spain.; 5 Rheumatology Department, Hospital del Mar, Barcelona, Spain.; 6 Servei de Reumatologia, Hospital de la Santa Creu i Sant Pau, Barcelona, Spain.; 7 Rheumatology Department, Hospital Universitari Mútua de Terrassa, Terrassa, Barcelona, Spain.; 8 Unit of Synthesis and Biomedical Applications of Peptides, Institute of Advanced Chemistry of Catalonia (IQAC-CSIC), Barcelona, Spain.

**Keywords:** Autoimmunity, Inflammation, Arthritis, Epigenetics

## Abstract

Identifying predictive biomarkers at early stages of inflammatory arthritis is crucial for starting appropriate therapies to avoid poor outcomes. Monocytes (MOs) and macrophages, largely associated with arthritis, are contributors and sensors of inflammation through epigenetic modifications. In this study, we investigated associations between clinical features and DNA methylation in blood and synovial fluid (SF) MOs in a prospective cohort of patients with early inflammatory arthritis. DNA methylation profiles of undifferentiated arthritis (UA) blood MOs exhibited marked alterations in comparison with those from healthy donors. We identified additional differences both in blood and SF MOs after comparing patients with UA grouped by their future outcomes, i.e., good versus poor. Patient profiles in subsequent visits revealed a reversion toward a healthy level in both groups, those requiring disease-modifying antirheumatic drugs and those who remitted spontaneously. Changes in disease activity between visits also affected DNA methylation, which was partially concomitant in the SF of UA and in blood MOs of patients with rheumatoid arthritis. Epigenetic similarities between arthritis types allow a common prediction of disease activity. Our results constitute a resource of DNA methylation–based biomarkers of poor prognosis, disease activity, and treatment efficacy for the personalized clinical management of early inflammatory arthritis.

## Graphical abstract

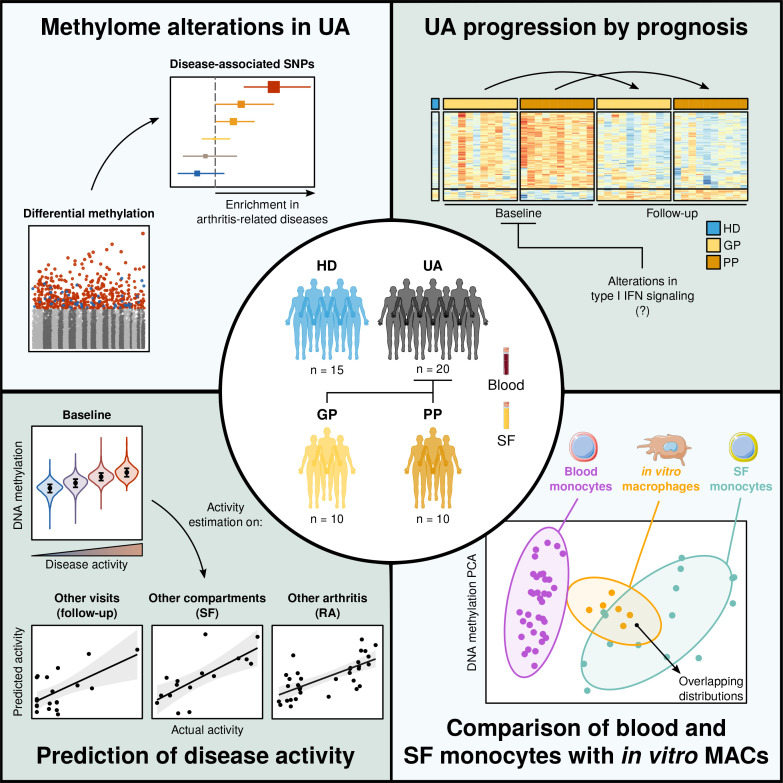

## Introduction

In early arthritis, a rapid identification of prognostic signs before the disease progresses to the establishment of aggressive or established forms can be highly beneficial for patients. Undifferentiated arthritis (UA) is an inflammatory form of the arthritis spectrum, in which patients cannot be diagnosed by definite disease classification criteria (1–3). A considerable proportion of patients with early UA eventually develop definite arthritis — rheumatoid arthritis (RA), psoriatic arthritis (PsA), or peripheral spondyloarthritis (pSpA), among others — while the rest either remain as UA or remit spontaneously, without the need for treatment with disease-modifying antirheumatic drugs (DMARDs; ref. 4).

In the past few years, identifying those patients with early arthritis with a higher probability of developing severe courses of the disease or worse responses to future therapy has become a major goal (5–7). The delay in DMARD administration for those patients who will eventually need it can lead to eventual erosive arthritis, resulting in joint damage, functional impairment, and poor quality of life (8). For this reason, there is a need to discover novel biomarkers that are highly specific to those cases so that a rapid, tailored intervention can be provided before the patient’s well-being deteriorates further.

DNA methylation is an epigenetic modification that acts as a dynamic mediator of the environment–genome interface, through which it has the potential to shape cell function and phenotype (9, 10). DNA methylation is frequently associated with the repression of gene expression, although their specific mutual relationship varies depending on the genomic context (11). To date, methylome alterations have been associated with a range of immune-mediated diseases (10, 12). Depending on the context, this association can be causal — either direct (13) or through intermediation of genetic susceptibility (14) — or a consequence of a pathogenic condition (15). This sensing capability makes DNA methylation especially useful for providing insights into the molecular alterations of both general conditions and individual patient features. For this reason, its application as a biomarker for personalized diagnosis is gaining in importance (7, 16–18).

Myeloid cells have been widely studied in inflammatory diseases because of their ability to sense and react to inflammatory cues in a variety of contexts (19, 20). Among others, one of the mechanisms through which these stimuli are integrated into their phenotype is DNA methylation (15, 21). For instance, monocytes (MOs) undergo methylome alterations in immune-mediated inflammatory diseases, such as RA, multiple sclerosis, systemic lupus erythematosus, and Crohn’s disease (15, 22–24). In addition, they are terminally differentiated cells, which allows an unbiased interpretation of their epigenetic features, which can be largely attributed to their immediate response to a particular context. For this reason, these cells pose as ideal candidates for evaluating patient molecular profiles through analysis of their epigenome.

In this study, we characterized the DNA methylation profiles of MOs from patients with UA and described their alterations when compared with healthy controls. Furthermore, monocytic populations were analyzed in the peripheral blood and the synovial fluid (SF), allowing for a comparative characterization of the 2 compartments. These data were further integrated with additional data of MO-derived macrophages (MACs), differentiated in vitro. Patients with UA were followed up in subsequent clinical visits, and the effects of prognosis (good vs. poor), treatment choice (DMARD vs. non-DMARD) and changes in activity were studied in relation to their methylome profiles. Our analyses allowed us to identify candidate biomarkers of poor prognosis in UA and monitor reversion of DNA methylation alterations in relation to treatment and remission.

## Results

### Early, treatment-naive UA blood MOs display DNA methylation alterations in immune-related regulatory regions.

**Figure 1:**
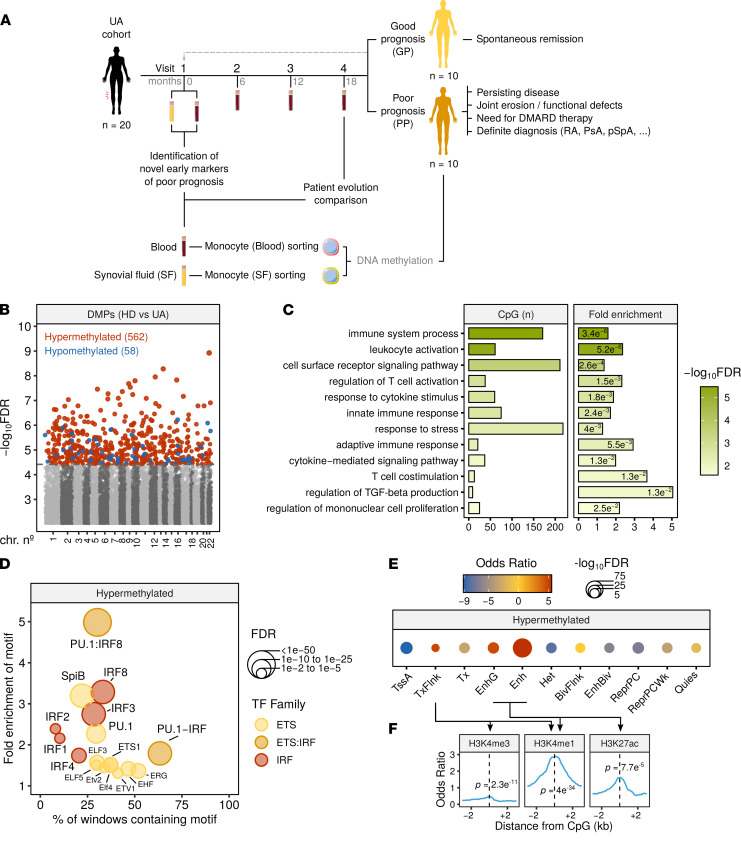
DNA methylation differences between UA and HD blood MOs. (**A**) Flowchart summarizing the cohort timeline characteristics and the analytical workflow. (**B**) Manhattan plot depicting differential methylation significance results, by autosome. Colored dots indicate significant DMPs (*limma* FDR < 0.05) between UA (*n* = 20) and HD (*n* = 15). Blue indicates hypomethylation in UA, and red indicates hypermethylation in UA, relative to HD. (**C**) Significant GO categories selected from the analysis with GREAT of the hypermethylated DMPs. The number of CpGs, fold enrichment, and hypergeometric test *P* value are depicted for every category. (**D**) Significantly enriched TF motifs in the hypermethylated cluster regions, identified by HOMER. (**E**) Chromatin functional state enrichment analysis of the hypermethylated DMPs on CD14 primary cells ChromHMM public data from Roadmap Epigenomics Project. (**F**) Enrichment of MO histone mark ChIP-Seq public data around the hypermethylated DMP coordinates. *P* values are derived from Fisher’s exact tests. Arrows specify which histone marks are contained in each of the chromatin state categories in **E**. TssA, active TSS; TxFlnk, transcript at gene 5′ and 3′; Tx, strong transcription; EnhG, genic enhancers; Enh, enhancers; Het, heterochromatin; BivFlnk, flanking bivalent TSS/Enh; EnhBiv, bivalent enhancer; ReprPC, repressed PolyComb; ReprPCWk, weak repressed PolyComb; Quies, quiescent.

We isolated blood MOs from treatment-naive patients with early UA to analyze DNA methylation. Samples from these patients were obtained at baseline (visit 1) and at follow-up visits scheduled every 6 months, for an average of 4 visits in total (Figure 1A and Supplemental Figure 1A; supplemental material available online with this article; https://doi.org/10.1172/jci.insight.158783DS1). Descriptive and clinical information of each sample, including age, sex, autoantibody seropositivity, and disease activity, were collected simultaneously (summarized in Supplemental Table 1 and detailed in full in Supplemental Table 2). A total of 20 patients with UA and 15 age- and sex-matched healthy donors (HDs) were analyzed (Supplemental Table 1A). The comparison of HDs and patients with UA (at baseline) revealed the presence of 620 differentially methylated positions (DMPs) between the 2 groups (FDR < 0.05). The DMPs identified were mainly hypermethylated in UA (562 DMPs, 91%), though some were hypomethylated (58 DMPs, 9%). They were homogenously distributed across the autosomes (Figure 1B and Supplemental Table 3).

Gene Ontology (GO) analysis showed the 620 DMPs to be significantly enriched in multiple functional categories related to both adaptive and innate immunity, response to stress, and cytokine signaling pathways (Figure 1C). We identified candidate genes of established relevance to the arthritis spectrum (Supplemental Figure 1B). Among these, we pinpointed FMS-like tyrosine kinase 3 (*FLT3*), the expression of which has been shown to be affected in blood MOs of patients with RA (25); *IFNGR2*, which encodes subunit 2 of the IFN-γ receptor; IFN-response factor 4 (*IRF4*), which is involved in the inflammatory polarization during dendritic cell differentiation from MOs (26); *IL12A*, *IL1RAP*, and *IL6ST*, which are associated with cytokine signaling in the context of immune cell activation; IL-1R–associated kinase 2 (*IRAK2*), the intermediary kinase in the TLR/IL-1R pathway; and TNF alpha–induced protein 3 (*TNFAIP3*), induced by TNF, which encodes a negative regulator of the TNF pathway. Additionally, a differentially methylated region was identified in the *TGFB2* locus, which spans 4 DMPs (Supplemental Figure 1C).

From this point onward, all analyses were exclusively performed on the hypermethylated DMPs (>90% of the total). Transcription factor binding motif enrichment (TFME) analysis of these DMPs revealed significant enrichment of motifs from the ETS (PU.1, SpiB, and ETS1, among others) and the IRF families (IRF1, 2, 3, 4, and 8; Figure 1D).

In addition, to contextualize the identified DMPs from a genomic standpoint, we analyzed the enrichment in previously characterized chromatin states, defined by combinations of distinct histone modification marks in blood MOs (27). The readout of this analysis makes it possible to characterize the putative impact of the alterations in particular regions upon gene expression. The DMPs analyzed were mostly enriched in enhancer regions, and, to a lesser extent, in transcript-flanking regions (Figure 1E). These results were supported using ChIP-Seq public data of histone marks from human MOs (see Methods). Signal distribution was plotted around the center of the DMPs, and enrichment was calculated at the DMP coordinate by a Fisher’s exact test (Figure 1F). Histone modifications contributing to the significantly enriched states (H3K4me3, H3K4me1, and H3K27ac; ref. 27) showed significant enrichment relative to background regions in all cases, reinforcing the results presented in Figure 1E.

We then attempted to ascertain whether the alterations associated with UA were in close proximity to genomic positions previously associated with susceptibility to arthritis (see Methods). Of note, a majority of SNPs associated with disease risk in GWAS are located in noncoding regulatory regions (28), similar to the DNA methylation alterations found in UA. Interestingly, UA-associated DMPs were found to be significantly close to SNPs previously associated with RA, juvenile idiopathic arthritis, and the anti-citrullinated protein Ab-positive (ACPA-positive) RA subtype (Supplemental Figure 1D). In summary, these results indicate that, in UA, dynamic DNA methylation mostly occurs in genomic regulatory regions related to the immune system function and potentially associated with the pathology of arthritis.

### DNA methylation alterations at baseline anticipate future prognosis.

The evaluation of patients over time enabled the classification of patients with UA into 2 distinct prognostic levels. The first group of patients (*n* = 10) was defined by an overall positive clinical and biological evolution, in which the disease achieves remission spontaneously or after treatment with an NSAID. The second group of patients (*n* = 10) was characterized by cases with a generally worse outcome than the first, in which severe arthritis persisted despite treatment with an NSAID, there was a functional impairment (as evaluated by the Health Assessment Questionnaire), or even a definite arthritis diagnosis of RA, PsA, or pSpA was reached (Supplemental Table 2). Patients in this second group required treatment with DMARDs before their final visit (visit 4) in 9 out of 10 cases, although 1 patient (UA14) repeatedly refused to undergo DMARD treatment throughout the follow-up. An additional patient did not require DMARDs after the first visit but presented a gain in functional disability at the last visit (UA12). Retrospectively, those groups were regarded as “good prognosis” (GP) and “poor prognosis” (PP), respectively (Figure 1A and Supplemental Table 1B). Of note, there were no significant differences in sex (*P* = 0.648, χ^2^ test) or age (*P* = 1, Wilcoxon’s test) distributions between both groups (Supplemental Table 1B).

**Figure 2:**
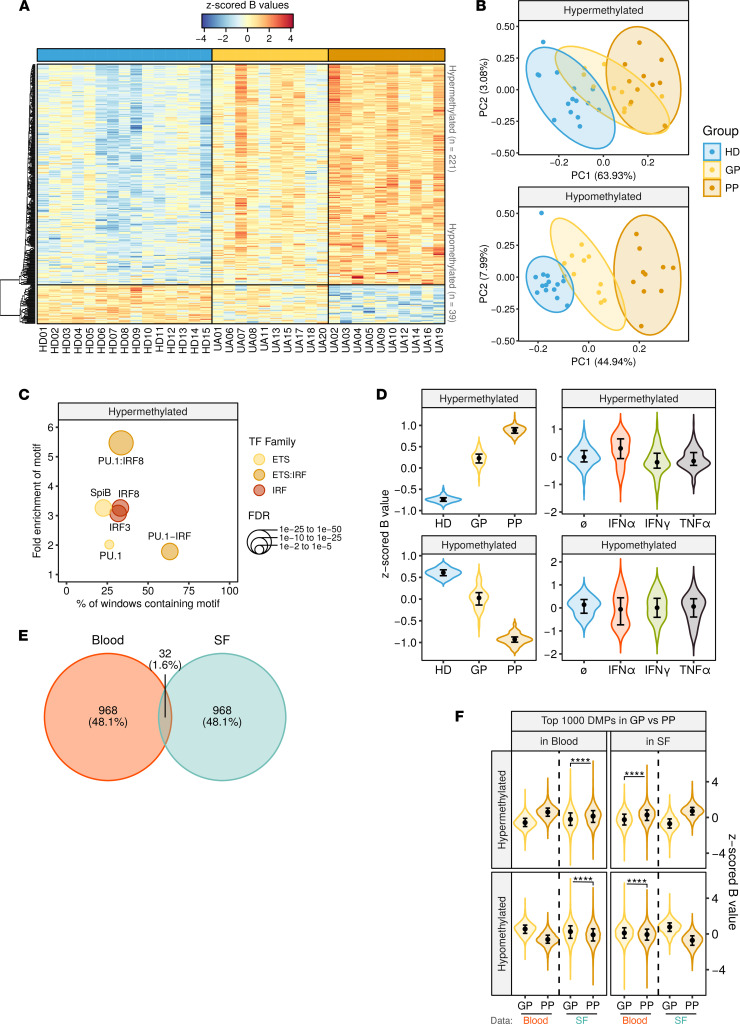
DNA methylation differences between GP, PP, and HD. (**A**) Heatmap showing DMPs (FDR < 0.05) between GP group (*n* = 10), PP group (*n* = 10), and HD group (*n* = 15). Blue and red indicate lower and higher methylation, respectively. (**B**) PCA of the DMPs in **A**. Ellipses show the 95% CI of the distribution of every sample group. (**C**) Significantly enriched TF motifs in the hypermethylated cluster regions, identified by HOMER. (**D**) Violin plots showing *z*-scored β values of the hypermethylated and hypomethylated clusters, in data from **A** and in public data from MOs purified after PBMC stimulation with cytokines for 4 days (*n* = 3). (**E**) Venn diagram showing overlap between the top 1000 most significant DMPs in the GP versus PP comparison, in blood (*n* = 10 patients in each group) and SF MOs (*n* = 8 patients in each group). (**F**) Violin plot showing the top 1000 most significant DMPs in GP versus PP comparison in blood and SF. The *x* axis indicates which data are contained in every violin plot, while column facets indicate the data set from which the top DMPs were selected. For the data sets not included in the DMP selection, differences in the medians were verified by a Wilcoxon’s test. ****P < 0.0001. In **D** and **F**, violin plots show density curves, and circles and vertical lines show the median and the 25th to 75th percentiles.

The comparison of the DNA methylation profiles at baseline of patients based on their future classification (GP, PP) and HDs identified 260 significant DMPs among the 3 conditions (Figure 2A and Supplemental Table 4). Of those, 221 were hypermethylated and 39 hypomethylated in patients with UA (FDR < 0.05). In a principal component analysis (PCA), these DMPs showed the most extreme overall methylation level in the PP group, while the GP group was situated between the HDs and the PP group, suggesting a cumulative degree of alterations (HD to GP to PP) within these regions (Figure 2B).

TFME analysis of the hypermethylated DMPs further revealed a presence of motifs from the ETS and the IRF families (Figure 2C), similar to what was observed in the comparison of HDs and patients with UA (Figure 1D). These results suggest a more pronounced activity of those pathways in the PP group, which shows the most distinct profiles.

To evaluate the hypothesis that inflammatory pathways might induce the PP signature through soluble cues in the peripheral blood, we analyzed our team’s previously published DNA methylation data corresponding to MOs purified from PBMCs after treatment with inflammatory cytokines (IFN-α, IFN-γ, and TNF-α) in vitro (15) and then inspected the methylation levels of both DMP clusters under these conditions. The tendency of PP in both DMP clusters was partially recapitulated by the stimulation with IFN-α, although the effect was more pronounced in the hypermethylated cluster (Figure 2D). These results are consistent with those of previous studies describing an IFN signature in patients with arthritis with poor outcomes (6).

We then checked correspondence of the obtained results in the synovial compartment by selecting the top 1000 most significant DMPs in the GP versus PP group comparison in each compartment (blood, SF) and calculating the overlap. The results in the 2 compartments displayed relatively low coincidence (32 DMPs, 1.6% of the total) (Figure 2E). Nevertheless, after unsupervised inspection of the overall tendency of the DMPs (GP vs. PP) identified in each compartment, we found a concomitant tendency of the average methylation levels of the same regions in the other compartment (Figure 2F). These results suggest the existence of common and independent effects of future outcomes at the synovial and peripheral levels.

### Synovial MOs display in vitro MAC epigenetic features.

**Figure 3:**
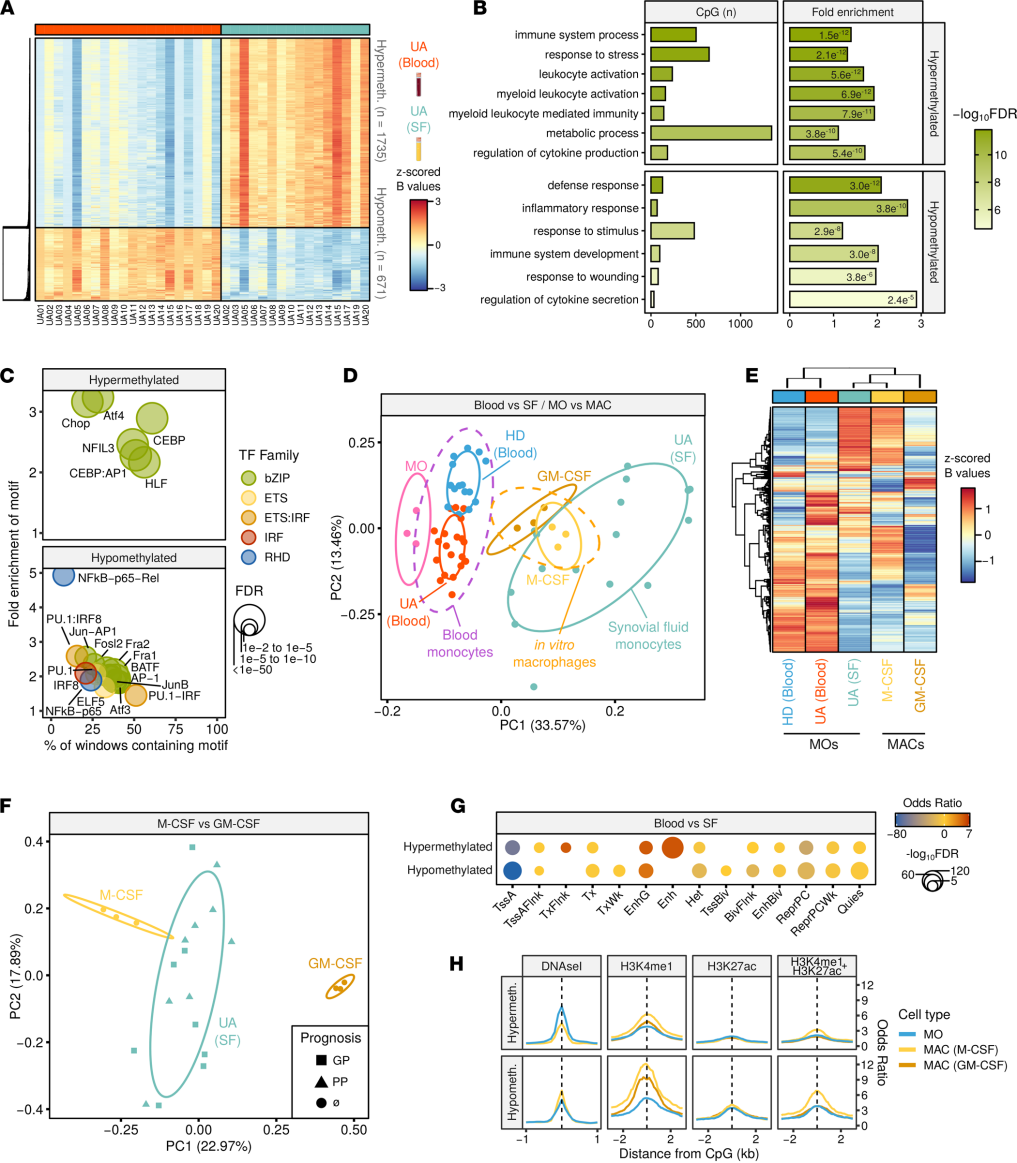
DNA methylation study in synovial MOs. (**A**) Heatmap showing DMPs between blood (*n* = 20) and SF (*n* = 16) MOs, paired by patient (FDR < 0.05, Δβ ≥ 0.15). (**B**) Significant GO categories selected from the analysis with GREAT of the DMPs. The number of CpGs, fold enrichment, and hypergeometric test *P* value is depicted for every category. (**C**) Significantly enriched TF motifs in the DMP regions, identified by HOMER. (**D**) PCA of the DMPs between the UA data set (HD, *n* = 15), UA blood (*n* = 20) and UA SF (*n* = 16), on one hand, and between conditions in the MAC in vitro differentiation data set (MO, *n* = 3), M-CSF (*n* = 3) and GM-CSF (*n* = 3), on the other (see Methods). (**E**) Heatmap of the DMPs in **D** for HD, UA blood, SF blood, M-CSF, and GM-CSF with hierarchical clustering. (**F**) PCA of the DMPs in the M-CSF versus GM-CSF. MAC subtypes and UA SF samples are displayed in different colors, and the prognostic group is indicated by shape. (**G**) Chromatin functional state enrichment analysis of the DMPs on CD14 primary cells public data from the Roadmap Epigenomics Project. (**H**) Enrichment of MO and MAC histone mark ChIP-Seq public data around the hypermethylated DMP coordinates. Cell types are indicated by colors. Arrows specify which histone marks are contained in each of the chromatin state categories in **F**. RHD, Rel homology domain; TssA, active TSS; TssAFlnk, flanking active TSS; TxFlnk, transcript at gene 5′ and 3′; Tx, strong transcription; TxWk, weak transcription; EnhG, genic enhancers; Enh, enhancers; Het, heterochromatin; TssBiv, bivalent/poised TSS; BivFlnk, flanking bivalent TSS/Enh; EnhBiv, bivalent enhancer; ReprPC, repressed PolyComb; ReprPCWk, weak repressed PolyComb; Quies, quiescent.

To further characterize the epigenetic profiles of UA MOs in peripheral blood (UA blood) and UA SF, we compared the DNA methylation profiles of MOs from the 2 compartments, matched by patient, and found major differences between them. Specifically, compared with blood MOs, SF MOs exhibited hypermethylation in 1735 DMPs and hypomethylation in 671 DMPs (FDR < 0.05 and an absolute difference in β value of 0.15; Figure 3A and Supplemental Table 5). These results are consistent with previous reports indicating that differences in the environment (e.g., cytokines, growth factors, etc.) influence the DNA methylation status in MOs (15) and that myeloid cells isolated from the SF are, in essence, MACs (29). These DMPs were enriched in GO categories related to immune system functions, cytokine signaling, and wounding response (Figure 3B).

TFME analysis of the hypermethylated DMPs revealed an enrichment of TFs from the basic leucine zipper (bZIP) domain family, including C/EBP and activating TF4 (ATF4), among others. The hypomethylated DMPs showed enrichment of TFs from the IRF (IRF8), ETS (PU.1, among others), bZIP (activating protein 1 [AP-1], complex-related Fos-related antigen 1/2 [Fra1/2], bZIP ATF-like TF [BATF], JunB, and Fosl2, among others), and NF-κB families (Figure 3C).

Given that most of these TFs have been involved in the differentiation of MACs from MOs in vivo (20) and in vitro (30), we compared blood and SF data to those of in vitro MO-derived MACs. The top 1000 DMPs between blood (UA and HD) and SF (UA) MOs, as well as between MOs and MACs, differentiated in vitro with GM-CSF (M1-type MACs) and M-CSF (M2-type MACs), were plotted in the first 2 principal components of a PCA (Figure 3D). The DNA methylation values in these positions revealed an overlap of the UA SF and MAC distributions, especially the M-CSF. This result was supported by the hierarchical clustering of those DMPs, in which the UA SF and both MAC subtypes were aggregated in a cluster (Figure 3E). To address the hypothesis that differential signatures might determine prognosis in SF MOs, we then focused on the differences between GM-CSF and M-CSF and then plotted UA SF data, highlighting the prognosis group (GP or PP). Once again, the UA SF distribution was more proximal to that of M-CSF than to GM-CSF, although we did not identify any distribution differences in association with the prognostic group (Figure 3F).

Finally, we performed a chromatin functional enrichment analysis, which again revealed an enrichment of the identified DMPs in MO enhancer regions, for the hypermethylated and hypomethylated regions (Figure 3G). This result was further confirmed by histone mark ChIP-Seq data, in which DMPs revealed a gain in active enhancer marks (H3K4me1 + H3K27ac) in M-CSF MACs (Figure 3H). This effect was more marked in the hypomethylated cluster, which concomitantly showed an increase in chromatin accessibility in M-CSF when compared with MO (Figure 3H). Taken together, these results suggest the existence of a MAC-like epigenomic profile in SF MOs in patients with UA.

### Methylome profiles of patients with UA revert over time to different extents in relation to the prognosis.

**Figure 4:**
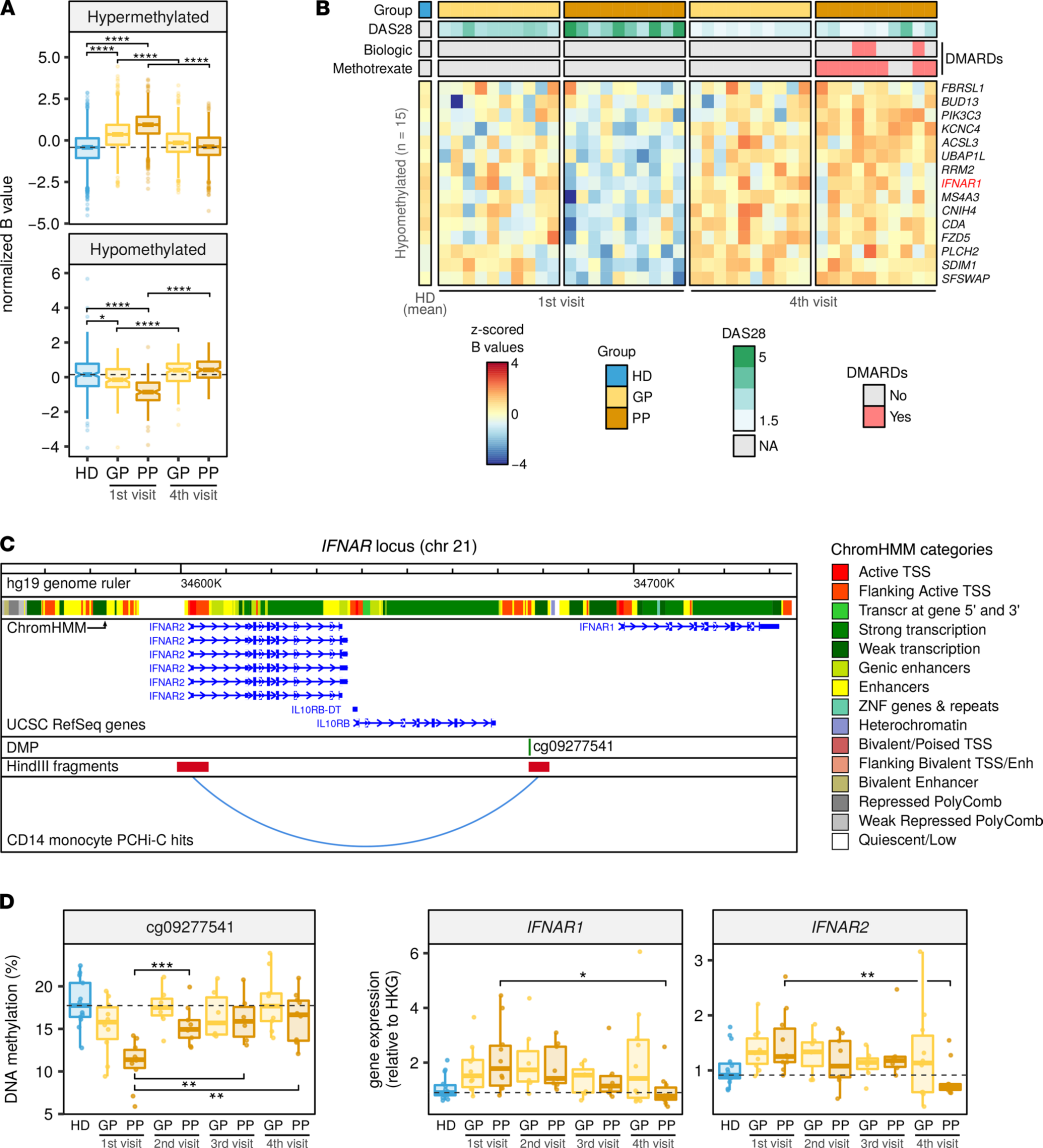
Evolution of DNA methylation profiles in subsequent visits. (**A**) Box plots showing *z*-scored β values of DMPs between the first and fourth visits, by GP and PP, paired by patient and using DAS28 as a covariate (FDR < 0.05). (**B**) Heatmap of the DMPs in the hypomethylated cluster. Group, DAS28, and treatment are shown for every patient at the top, and the respective legend scales are shown to the right of the heatmap. Blue and red indicate lower and higher methylation, respectively. (**C**) *IFNAR* locus with PCHi-C interaction public data. (**D**) DNA methylation of cg09277541 (left panel) and gene expression of *IFNAR1* and *IFNAR2* (right panel) in visits 1–4, by prognosis group. DMP and interacting HindIII fragments are shown below a genome browser annotation of transcripts and MO ChromHMM tracks (see Methods). DNA methylation and gene expression from **D** were analyzed by bisulfite pyrosequencing and qRT-PCR, respectively. *RPL38* was used as the HKG. The number of samples analyzed for each group in every time point is indicated in Supplemental Figure 1A. In **A** and **D**, each box represents the 25th to 75th percentiles. The lines inside the boxes represent the median. The lines outside the boxes represent the 25th percentile minus 1.5 times the IQR and the 75th percentile plus 1.5 times the IQR. Pairwise group differences were evaluated by 2-tailed Wilcoxon’s tests. *P < 0.05, **P < 0.01, ***P < 0.001, ****P < 0.0001.

To evaluate the effect of disease progression on DNA methylation, we compared the profiles of patients with UA at baseline with those obtained after follow-up visits. In particular, we analyzed the last visit in which patient blood samples were collected (visit 4, 18 months after visit 1; Figure 1A). The profiles from the first and fourth visits were compared, paired by patient, in both prognosis groups (Figure 4A). Patient disease activity, measured by disease activity score 28 (DAS28) (see definition in Methods), was used as a covariate to account for the differences caused by variation in activity between the 2 visits (Supplemental Table 1, D and E, and Supplemental Figure 2A). The comparison between visits revealed 250 hypermethylated and 15 hypomethylated DMPs in the first visit of the PP group (FDR < 0.05) (Supplemental Figure 2B and Supplemental Table 6). In the hypermethylated cluster, the GP and PP groups were both altered in the first visit, in a cumulative fashion, while on the fourth visit, their average methylation had reverted to levels comparable to that of the HD group. In the case of the hypomethylated cluster, DNA methylation alterations were slightly more specific to the PP group, since the HD and GP groups displayed more similar average methylation levels. Similar to the hypermethylated cluster, at the time of the fourth visit, both groups underwent a reversion in their average values, although the effect was more pronounced in the PP group, which experienced a higher range of reversion (Figure 4A). Interestingly, at visit 4, the reversion in the average DNA methylation levels of GP and PP was not correlated with their average activity levels (Supplemental Figure 2A), suggesting an activity-independent reversion of patient molecular alterations. The hypomethylated cluster tendency particularly discriminated the PP group, for which reason the DMPs in this cluster were considered robust biomarkers of poor prognosis. The DNA methylation values, DAS28, and DMARD treatment of every patient are depicted in Figure 4B and Supplemental Figure 2B.

Considering that changes in DNA methylation mostly take place in distant regulatory regions rather than in genic promoters (31), we sought to investigate the effect of methylation alterations on mRNA transcription by inspecting 3D chromatin conformation data using the promote-capture Hi-C (PCHi-C) technique. PCHi-C assays allow the identification of the putative functional relationships between pairs of genomic regions that display a spatial physical interaction. In this technique, 1 end of the interacting pair is a gene promoter; thus, any molecular alterations in the partner of the pair are proposed to have a potential effect on the gene with the interacting promoter (32). PCHi-C data of blood MOs were integrated with the DMPs identified in Figure 4A (see Methods). Examples of long-range interactions overlapping with a DMP from Figure 4A and previously associated with susceptibility to arthritis (33–35) are depicted in Figure 4C and Supplemental Figure 2, C and E. DNA methylation and gene expression of the DMP-gene pair were concomitantly measured by bisulfite pyrosequencing and quantitative reverse transcription PCR (qRT-PCR), respectively. In this analysis, the entire series of samples from all 4 visits was included (Figure 4D and Supplemental Figure 2, D and F). Results from this analysis revealed a reversion over time of the DNA methylation profile of the selected genes toward HD levels, especially pronounced in the PP group, which was concomitant with the mRNA expression of genes in the interacting loci. Reversion in DNA methylation was already significant between visit 2 (Figure 4D) and visit 3 (Supplemental Figure 2, D and F). In most cases, at visit 4, the expression of the interacting genes in the PP group reverted to levels beyond HD, suggesting a further reduction in the immune activity of those patients. The trend between DNA methylation and gene expression in the cg09277541/*IFNAR* pair was inverse, while the trends for cg11193201/*FCGR2A* and cg02421308/*IL4R* were positive. This result could be possibly explained by the 3 DMPs overlapping functionally distinct genomic localizations, characterized by different combinations of epigenetic modifications (Figure 4C and Supplemental Figure 2, C and E, ChromHMM track), which may have disparate effects upon expression of the interacting gene (36).

### UA disease activity can be inferred from DNA methylation in blood and synovial MOs and in MOs from patients with RA.

**Figure 5:**
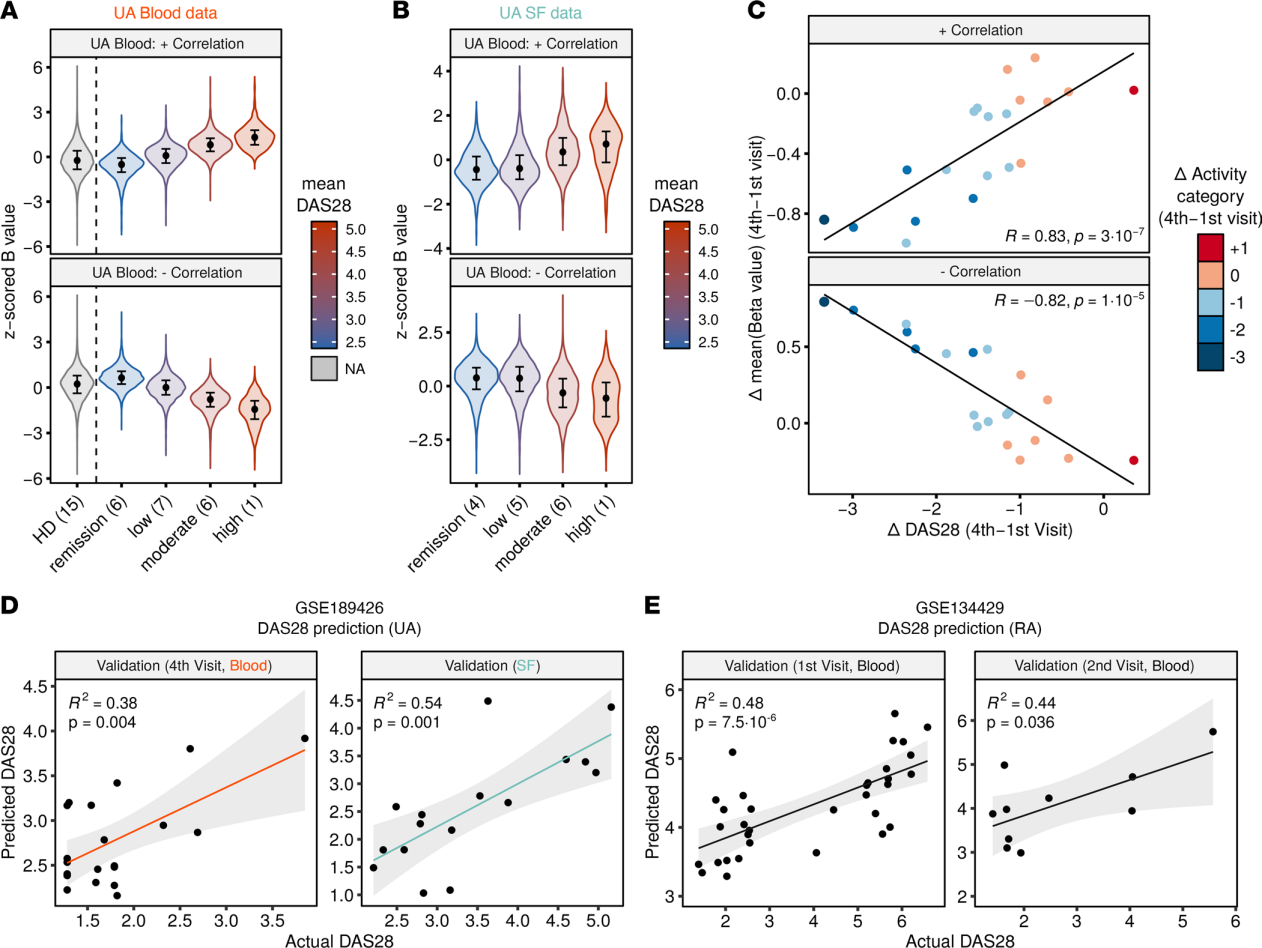
Correlation of DNA methylation and DAS28 in blood and SF of UA. (**A**) Violin plots showing *z*-scored β values of DAS28-correlated CpGs (*P* < 0.001, *ρ* ≥ 0.7), by activity category, in blood MOs. (**B**) Violin plots showing *z*-scored β values of DAS28-correlated CpGs in blood, by activity category, in SF MOs. Color in **A** and **B** indicates mean DAS28 score of each group. The number of samples in every activity category is noted in parentheses. (**C**) Scatter plots showing the correlation between the Δ of DAS28 and the Δ of *z*-scored β values between the first and fourth visits, in blood. Color indicates changes in activity categories. (**D**) Linear regression prediction of DAS28 from DNA methylation. First-visit blood samples were used to train the model, and prediction was performed on fourth-visit blood samples and first-visit SF samples. (**E**) Linear regression prediction of DAS28 on public data of MO samples from patients with RA, at first and second visits, after follow-up. In **D** and **E**, correlation coefficients (*R*^2^) and *P* values were calculated by Pearson’s correlation. Activity categories are defined as follows by the DAS28 value: remission (<2.6), low activity (2.6–3.2), moderate activity (3.2–5.1), and high activity (>5.1). In **A** and **B**, violin plots show density curves, and circles and vertical lines show the median and the 25th to 75th percentiles. The number of samples in **D** and **E** is indicated in Supplemental Figure 3G. In **D** and **E**, gray shades indicate the 95% CI of the value distributions.

To identify CpGs associated with disease activity, we calculated the Spearman’s correlation coefficient of the DAS28, namely, the DAS28–C-reactive protein (DAS28-CRP) and the genome-wide methylome profiles of all first-visit patient blood MO samples (correlation coefficient *ρ* cutoff = 0.7, *P* cutoff = 1 × 10^–3^). DNA methylation distributions of the correlated CpGs in every activity category (remission, low, moderate, and high; see Methods) are depicted in Figure 5A. HD distributions are shown in parallel, in an unsupervised manner. It is of note that the correlated CpGs had no major overlap with the prognosis-associated DMPs identified in Figure 2A and Supplemental Figure 3A, reinforcing the notion of a methylation-based good and poor prognosis classification that is independent of disease activity. To check for a translation of blood MO results to SF MOs, we inspected the blood-correlated CpGs in the SF MO data. The correlative tendency of the activity categories was recapitulated in the SF MOs (Figure 5B). Independently, the correlation was replicated in the SF data, and the results in both compartments were tested for concordance using a Fisher’s exact test (Supplemental Figure 3, B and C). The correlation in both compartments was significantly concurrent for the positively and negatively correlated CpGs (Supplemental Figure 3C). There were more significant CpGs that were negatively correlated with DAS28 (61% in blood, 56% in SF), and these overlapped more frequently between the 2 data sets (75% of CpGs correlated in blood+SF; log_2_ odds ratio = 6.2, *P* = 2.1 × 10^–55^; Supplemental Figure 3, B–D). Examples of CpGs correlated with DAS28 in both blood and SF are illustrated in Supplemental Figure 3E.

Since epigenetic modifications are well-known reversible biological features, we decided to evaluate the reversibility of DNA methylation levels with respect to variations in DAS28 in subsequent clinical visits (Supplemental Figure 3F). To this end, we calculated the Δ in mean DNA methylation (of the previously identified DAS28-correlated CpGs) and the Δ of DAS28 between the first and fourth visits, for every patient, and performed a Spearman’s correlation (Figure 5C). The great majority of cases (19/20) experienced a decrease in activity between visits, except for 1 patient, who was not initially prescribed with DMARD therapy (UA12) throughout this period (Supplemental Figure 3F) and experienced an increase in the activity category. For both the positively and negatively correlated CpGs, there was a significant correlation between the change in mean methylation and the change in DAS28. Moreover, patients who experienced a shift in more activity categories also underwent a greater change in mean methylation between the 2 visits, highlighting the importance of the activity category in addition to the numerical DAS28 value (Figure 5C).

Next, we tested the applicability of the previous finding by generating predictive estimates of disease activity in independent samples. To this end, we trained a multivariate linear regression (MLR) model with DNA methylation data from first-visit samples, with DAS28 as the independent variable. The model was validated using MO DNA methylation data from samples of patients with UA at the follow-up visit (fourth visit) and in the SF (Supplemental Figure 3G, top row), where models including sequential numbers of CpGs (from 1 to 7, see Methods) were tested. Models with 3 CpGs performed best on average, in an internal 5-fold cross-validation and in both UA validation data sets (Figure 5D and Supplemental Table 9), consistent with previously proposed MLR models to estimate the DAS28 in MOs of patients with RA (15).

Given that DAS28 is considered a standard disease activity measure for both UA and RA, we decided to establish commonalities of the effect of DAS28 on DNA methylation between the 2 entities. Upon inspection of the top 100 DAS28-correlated CpGs in UA and RA, respectively, we verified the existence of a similar trend in those CpGs between patients from both diseases (Supplemental Figure 3H). We then applied the previously defined MLR model to predict activity values of the samples of patients with RA. The UA-derived model predicted DAS28 in 2 RA cohorts (first visit and follow-up second visit; Supplemental Figure 3G) with significant accuracy (Figure 5E). Overall, these results imply that disease activity can be predicted using a common approach in cells from distinct physiological compartments and in distinct types of arthritis.

## Discussion

Our results represent an invaluable resource for the application of the identified DNA methylation dysregulation in early arthritis to uncover prognosis, progression, and impact of the treatment. We have obtained, for the first time to our knowledge, the synovial and blood MO methylomes from patients with UA. Our results are in line with those of previous studies of inflammatory diseases in which DNA methylation changes in MOs have been described (15, 22–24). The identified alterations preferentially occurred in MO regulatory regions that are functionally related to immune cell function and inflammation. A significant number of these positions were found to be located near genomic regions that were previously associated with susceptibility to arthritis in GWAS. Hence, we propose that genetic and epigenetic variations in those positions may influence the pathological phenotype by affecting common pathways associated with disease.

The comparison of patients based on their future outcome evaluation revealed additional alterations between GP and PP patients, with an additive tendency when compared with the healthy group (HD–GP–PP). These prognosis-associated changes were shown to be partially recapitulated in vitro in MOs purified after stimulation of PBMCs with the cytokine IFN-α. IFN signatures have recursively been linked to poor prognosis or bad responses to treatment in arthritis such as RA (6, 37). Although the exact mechanism by which a DNA methylation IFN signature is acquired by patients with UA with worse future outcomes is not known, we believe it is possible that either an acute or a continuous effect of elevated circulating amounts of type I IFN could contribute to severe prognosis or to the faster differentiation of the disease. Actual efforts are set among our team and other research groups to confirm this mechanism and expand the findings in future studies with larger sample sizes. However, at this point, we cannot rule out the possibility that there is an underlying genetic predisposition toward a bad outcome in the PP group and that this may have an additional effect on DNA methylation.

When compared with blood MOs, the methylome profiles of the SF MOs showed a similar trend in the response to the prognostic group, although there were also a considerable number of changes that were nonoverlapping between the 2 compartments. This result suggests the existence of both common and independent mechanisms between blood and SF MOs underlying the sensitivity to the prognostic status. A possible explanation for this is that SF MOs (differentiated, MAC-like) and blood MOs (naive, MO-like) are essentially distinct cell types, which display different reactive capabilities driven by the expression of disparate repertoires of surface receptors (38). Also, differences between the inflammatory microenvironment at the synovium and the peripheral blood are likely to have an influence in the disparity of results from the 2 compartments. In fact, there is evidence for divergent phenotypic profiles between the blood and the synovial compartments, among subtypes of early RA with distinct prognosis (39).

The subsequent evaluation of patients in future visits revealed a reversion of the UA- and prognosis-associated methylome profiles. That effect was more pronounced in the PP group, which after treatment with DMARDs reached a level comparable to that of naturally remitting GP patients and similar to — or even exceeding — that of healthy controls. Also, to study the potential effect of DNA methylation alterations upon gene expression, we made use of 3D chromatin conformation data. In addition to the epigenetic dynamism, gene expression analysis of the interacting genes unveiled a concomitant reversion to a healthy-like profile in both groups. This tendency generally preceded the changes in activity in successive visits, further reinforcing the notion that molecular alterations anticipate clinical features in patients with UA (5).

The previously described association between DNA methylation and disease activity (15) was confirmed in the present study. Our results allowed us to derive a predictive model to estimate the activity in subsequent visits and in other physiological compartments, such as the SF, which showed a similar effect to that of the activity identified in peripheral blood. This finding highlights the reliability of peripheral blood MOs as bona fide sensors of systemic inflammation. The predictive model also yielded significant results when applied to blood MO samples from patients with RA (Figure 5E). These findings lead us to hypothesize the existence of a reversible, activity-associated DNA methylation signature that potentially acts through a common mechanism, independently of compartment or disease entity. We hope that it will be possible to identify a similar mechanism in other inflammatory diseases with poor disease activity indicators to assist clinicians in the assessment of prognosis factors and severity status of the disease.

Overall, these results suggest that the MO phenotype can be interpreted as a readout of the pathological milieu that is present in the blood of patients with UA, and that the results of its analysis can be leveraged to provide both descriptive and predictive conclusions about a particular systemic disease.

## Methods

### Patient cohort.

A total of 20 blood and 16 paired SF samples from patients with UA were obtained from the outpatient clinic of the Department of Rheumatology at the Hospital Clinic de Barcelona and 4 other university hospitals from the Barcelona area: Hospital Santa Creu i Sant Pau, Hospital del Mar, Hospital Parc Taulí (Sabadell), and Hospital Mútua de Terrassa (Terrassa). Patient samples were collected at baseline (visit 1) and correspond to patients with no prior treatment with DMARDs (including antimalarial agents) or glucocorticoids at a dose higher than 5 mg of prednisone per day and who did not meet the 2010 American College of Rheumatology/European League Against Rheumatism (ACR/EULAR) criteria (1) for RA, the Classification for Psoriatic Arthritis (CASPAR) criteria (2) for PsA, or the ASAS Assessment of Spondyloarthritis International Society (ASAS) criteria for pSpA (3). The clinical data of the patients included in the study are summarized in Supplemental Table 1A. Additional blood samples from 15 HDs were obtained, matched by age and sex (Supplemental Table 1A). Patient samples were also collected during 4 follow-up visits at 6-month intervals (Figure 1A and Supplemental Figure 1A). Patient activity was measured with DAS28, a composite score derived from 4 measurements: the number of tender joints (in 28 consensus joints), the number of swollen joints (in the same 28 joints), the concentration of CRP in the blood serum, and an assessment of general health by the patient. Activity categories are defined as follows by the DAS28 value: remission (<2.6), low activity (2.6–3.2), moderate activity (3.2–5.1), and high activity (>5.1; ref. 40). After the final visit (18 months), patient prognosis was defined as negative when any of the following conditions, as defined by the clinician, were met: a) persisting disease, b) joint erosion or functional impairment, c) need for DMARD therapy, or d) definite arthritis diagnosis. The remaining patients remitted spontaneously (without DMARD therapy) before their fourth visit (after 18 months), and so their diagnoses were regarded as positive. Based on those evaluations, patient samples at the baseline visit were designated as GP and PP, respectively (Supplemental Table 1B and Figure 1A). The size of the patient cohort was determined by the challenge of our restrictive inclusion criteria — early and untreated patients with UA. Indeed, we reached 20 included patients after the collaboration of other university hospitals from our area.

### Isolation of blood and SF MO populations.

SF samples were diluted in PBS solution and thoroughly resuspended through a serological pipette to ensure proper homogenization. Fresh blood and homogenized SF samples were diluted in PBS and centrifuged in a Ficoll-Paque gradient to isolate mononuclear cells. Mononuclear cells of both compartments were processed in parallel, and the MO population was isolated by FACS, following the previously described protocol (15).

### DNA methylation data generation.

Infinium MethylationEPIC BeadChips arrays (Illumina) were used to analyze DNA methylation. MO genomic DNA was extracted, modified, and analyzed on arrays by the procedure described (5). Inferential analyses and data visualization were performed on M and β values (41), respectively. Distributions of β values are heteroskedastic and, thus, not recommended for statistical purposes (41). New DNA methylation data presented in this paper are deposited in the National Center for Biotechnology Information’s (NCBI) Gene Expression Omnibus (GEO) database (GSE189426 and GSE189422).

### Quality control, data normalization, and differential methylation analysis.

DNA methylation data quality control and exploratory and inferential analyses were conducted with the *shinyÉPICO* application (42), which relies on functions from the *minfi* (43), *lumi* (44), and *limma* (45) R libraries, among others. Probe detection was determined by a cutoff value of *P* < 0.01 and normalization of the raw methylation values was performed using the Noob method followed by the Quantile method. CpGs coinciding with an SNP locus were excluded from the analysis. Additionally, sex chromosomes (X and Y) were excluded from the analysis to avoid data discordancy between samples. Significant DMPs with respect to groups were identified using an empirical Bayes-moderated *t* test method with defined empirical array weights (46), included in the *limma* package (45). For the DMPs depicted in Figures 1, 2, and 4, the only threshold applied was an FDR less than 0.05. In Figures 1, 2, and 4, the cutoff for the difference of DNA methylation was an absolute Δβ greater than 0, and, therefore, hypermethylated genes are those for which the β value in UA is higher than in HDs and the other way around for those hypomethylated. In Figure 3, thresholds of FDR less than 0.05 and an absolute β value difference (blood minus SF) of 0.15 were used. DMPs illustrated in Figures 3 and 4 were identified in a paired fashion, including the patient as a covariate. Additionally, to remove the effect of the activity, the individual DAS28 value was also used as a covariate in the analysis of the data illustrated in Figure 4.

### Functional analyses: GO, motif enrichment, and chromatin features.

GO analysis was performed with the Genomic Regions Enrichment of Annotations Tool (GREAT; ref. 47) in the basal plus extension mode, using the *rGREAT* package in R. GO categories with an FDR less than 0.05 in the hypergeometric test were considered significantly enriched.

TFME analysis was conducted with HOMER software (48). A flanking window of 250 bp was added to the DMP coordinates before calculating the enrichment. The most highly enriched TFs are shown in the figures.

Chromatin functional state enrichment of the identified DMPs was analyzed using public CD14 primary cells data taken from the NIH Roadmap Epigenomics Project (http://www.roadmapepigenomics.org) generated with the ChromHMM software (49). A core 15-state model — primary HMM **—** constructed with data from 5 histone modification marks was used for this analysis. Enrichment and significance were estimated by Fisher’s exact tests. Significantly enriched states are shown in the figures.

MO and M-CSF MO-derived MAC genome-wide chromatin accessibility (DNaseI) and histone modification data (H3K4me1 and H3K27ac) were downloaded from the Blueprint consortium database (http://dcc.blueprint-epigenome.eu). GM-CSF MO-derived MAC histone modification data (H3K4me1 and H3K27ac) were obtained from a previous study (50). All these data were used to calculate enrichments around the DMP coordinates in 100 bp windows for the entire indicated ranges around the DMP (1 or 2 kb). Odds ratios and significance values were estimated with a Fisher’s exact test, and visual representations were produced using in-house R functions.

For all the aforementioned analyses, the EPIC array annotation was used as the background for calculating the enrichments.

### Heatmaps, PCA, and plots.

DMP heatmaps were generated using functions from the *gplots* and *ComplexHeatmap* R packages. Heatmap row-ordering was carried out using average-linkage hierarchical clustering. In all cases, red and blue indicate high and low normalized levels of DNA methylation, respectively. PCA coordinate matrices were obtained with the *prcomp* function, and PC-pair representations were plotted using functions in the *ggfortify* package in R. The heatmap in Figure 3A was derived from β values corrected for the patient covariate with the *removeBatchEffect* function from the *limma* package. Manhattan, bar, violin, bubble, line, box, scatter, and tile plots were generated using functions available in the *ggplot2* and *ggpubr* packages.

### SNP enrichment analysis.

SNPs associated with disease in GWAS were downloaded from the GWAS Catalog (51) (https://www.ebi.ac.uk/gwas). The following traits, including diseases from the arthritis spectrum and others (mostly non-inflammation-mediated diseases, used here as a negative control) were queried in the database: “Psoriatic arthritis,” “Ankylosing spondylitis,” “Rheumatoid arthritis,” “Rheumatoid arthritis (ACPA-positive),” “Rheumatoid arthritis (ACPA-negative),” “Osteoarthritis,” “Myocardial infarction,” “Schizophrenia,” “Osteoporosis,” “Juvenile idiopathic arthritis”, “Type 1 diabetes,” “Alzheimer’s disease,” and “Systemic lupus erythematosus.” SNPs with a significantly associated risk allele were subsetted, and the coincidence between the SNP coordinate and a 1 Mbp window around the DMP coordinate was calculated. Estimates of the enrichment (odds ratio) and its significance were computed with a Fisher’s exact test, using the EPIC array annotation as background regions. *P* values of every enrichment statistics were adjusted using the Benjamini-Hochberg method.

### Differentiation of MACs from peripheral blood MOs.

We obtained buffy coats from healthy, anonymous donors through the Catalan Blood and Tissue Bank (CBTB). The CBTB follows the principles of the World Medical Association Declaration of Helsinki. Before providing the first blood sample, all donors received detailed oral and written information and signed a consent form at the CBTB. PBMCs were isolated by Ficoll-Paque gradient centrifugation. MOs were isolated from PBMCs using positive selection with MACS magnetic bead–coupled CD14 Ab (Miltenyi Biotec catalog 130-050-201). Purified MOs were cultured in RPMI Medium 1640 + GlutaMAX (Gibco, Thermo Fisher Scientific) containing 10% FBS, 100 units/mL penicillin and 100 μg/mL streptomycin. For M-CSF and GM-CSF MAC differentiation, the medium was supplemented with 25ng/mL M-CSF (Peprotech) and 10ng/mL of GM-CSF (Peprotech), respectively. MOs were differentiated to MACs for 5 days.

### Joint analysis of MO and MAC data.

Data from GSE189426 and GSE189422 were quality controlled and analyzed jointly using the previously described methods. Since both data sets were analyzed in different batches, technical bias was removed with the *ComBat* function contained in the *sva* R package. After *ComBat*, differential methylation was performed and to further avoid batch-associated biases, only intra-batch comparisons were performed. Comparisons were conducted with *limma* between the following conditions: HD versus UA blood; UA blood versus UA SF; MO versus GM-CSF; MO versus M-CSF; and M-CSF versus GM-CSF. The top 1000 most significant DMPs in any of the comparisons were selected, and β values were scaled (*z*-scored) in every independent batch. PCA and heatmaps in Figure 3, D and E, were performed on the scaled β values of the selected DMPs. In Figure 3F, only the top 1000 most significant DMPs in M-CSF versus GM-CSF comparison were used.

### Integration and visualization of methylation and PCHi-C data.

We obtained previously generated PCHi-C data from healthy peripheral blood MOs (36). The PCHi-C technique and bioinformatic analysis of the obtained data are described (52). Significant PCHi-C interaction coordinates were overlapped with DMP coordinates using functions available in the *GenomicRanges* R package (53). Fragments mapping on a TSS on 1 end of the interaction were filtered, and arthritis-related genes were selected based on bibliography. Selected interactions were visualized using the WashU Epigenome Browser (54). The following tracks are shown, from top to bottom: reference genome (hg19 assembly), CD14 primary cells ChromHMM, UCSC RefSeq mRNAs, DMP coordinate, interacting genomic fragments obtained after digestion with HindIII enzyme (52), and MO PCHi-C interaction hit.

### Bisulfite pyrosequencing.

Genomic DNA was converted using the EZ DNA Methylation Gold kit (Zymo Research). PCR was performed using the bisulfite-converted DNA as input and primers were designed for each amplicon (Supplemental Table 8) using the PyroMark Assay Design 2.0 software (QIAGEN). PCR amplicons were sequenced using the PyroMark Q48 system and analyzed with PyroMark Q48 Autoprep software.

### qRT-PCR.

Total RNA was subjected to reverse transcription with the Transcriptor First Strand cDNA Synthesis Kit (Roche) following manufacturer’s instructions. qRT-PCR was performed in 3 technical replicates using LightCycler 480 SYBR Green Mix (Roche) and 5 ng of cDNA per replicate. The ΔΔCt method was used to determine the relative quantities of target genes, and *RPL38* was used as a housekeeping gene. qRT-PCR primers for the selected genes are described in Supplemental Table 8.

### Predictive regression models.

First, Spearman’s correlation coefficients between DNA methylation data of UA blood MOs from the first visit and DAS28 were calculated. Then, all the significantly correlated CpGs (correlation coefficient *ρ* cutoff = 0.7, *P* < 0.001) were used to build a linear model with DAS28 as the response variable, using the base R *lm* function. All significant coefficients in the resulting model were ordered by relative importance (lmg metric) with the help of functions available in the *relaimpo* R package (55). Starting from this ranked importance, the ideal combination of predictor CpGs (*n* = 7) was identified by a stepwise selection algorithm, implemented in R’s *step* function, the direction parameter being set to “both,” thereby enabling forward and backward stepwise model selection. Autocorrelation was discarded (Pearson’s *r* < 0.5) between all possible pairs of the finally selected 7 CpGs. Models spanning from 1–7 CpGs were then used to predict DAS28 a) in all discovery cohort samples (blood, visit 1); b) in 5-fold cross-validations within the discovery cohort samples; c) in visit 4 samples; and d) in SF samples. The coefficient of determination (*R*^2^) was calculated for all models, and the model containing 3 CpGs was selected for its best average performance among all the predicted data sets.

### Data sharing.

Methylation array data for this publication have been deposited in NCBI’s GEO database and are accessible through GEO Series accession number GSE189426.

### Statistics.

Unpaired 2-tailed Wilcoxon’s tests were used to compare median distributions among groups. Fisher’s exact tests were used to calculate enrichments of the DMPs over public ChIP-Seq data and disease-associated SNP coordinates, and to evaluate the concordance between blood and SF for the correlation of DNA methylation and activity. Activity and DNA methylation were correlated by applying a Spearman’s correlation. Prediction accuracy was evaluated by the coefficient of determination (*R*^2^) and the *P* value, computed by Pearson’s correlation. All significance values were computed with functions from R’s *stats* package and adjusted by the FDR method, when indicated. Significance values were summarized as follows: *P < 0.05, **P < 0.01, ***P < 0.001, ****P < 0.0001.

### Study approval.

The Committee for Human Subjects of the Hospital Clinic (Barcelona) approved the study (HCB2017/0562), which was conducted in accordance with the ethical guidelines of the 1975 Declaration of Helsinki. All samples were in compliance with the guidelines approved by the local ethics committee and all donors signed the informed consent form after they received oral and written information about the possibility that their blood would be used for research purposes.

## Author contributions

CCF, JRU, JDC, and EB conceived the experiments; CCF, LC, and IH performed the experiments; CCF and JRU conducted the bioinformatic analyses; CCF did the statistical analyses; JR, RC, ABA, AC, EG, CPG, GS, CDT, JAGP, RS, and JDC selected the patients, provided samples, and analyzed the data; CCF, JRU, JDC, and EB analyzed the data; CCF produced the graphical representations; and CCF, JRU, JDC, and EB wrote the paper. All authors read and approved the final manuscript.

## Supplementary Material

Supplemental data

Supplemental table 1

Supplemental table 2

Supplemental table 3

Supplemental table 4

Supplemental table 5

Supplemental table 6

Supplemental table 7

Supplemental table 8

Supplemental table 9

## Version 1 03/24/2022

In-Press Preview

## Version 2 05/09/2022

Electronic publication
