# Supplementary material for: The synovial and blood monocyte DNA methylomes mirror prognosis, evolution, and treatment in early arthritis
Source: JCI Insight. 2022 May 9;7(9):e158783. doi: 10.1172/jci.insight.158783 (PMC9090240; doi:10.1172/jci.insight.158783)

SUPPLEMENTARY FIGURE LEGENDS

Supplementary Figure 1. Design of the study and relationship to genetics. (A) Tile plot showing the number of patient samples available in each compartment (blood and synovial fluid, SF) at every visit. **(B)** B values of selected DMP examples. Violin plots show density curves, and circles and vertical lines show the median and the 25th to 75th percentiles. **(C)** DMR at the *TGFB2* locus. **(D)** Plot with the enrichment of disease susceptibility-associated SNPs (publicly available, see Methods) and DMPs in the HD *vs.* UA comparison, calculated by a Fisher's exact test. Text color indicates the type of disease ("arthritis" or "other disease"), square and whisker colors indicate the odds ratio (OR), and the square size indicates significance. Whisker ranges indicate 95% confidence intervals. Significantly enriched diseases are highlighted in black. ACPA, anti-citrullinated protein antibodies; FDR, false-discovery rate.

Supplementary Figure 2. DNA methylation changes over time and relationship with genes (A). Boxplots showing DAS28-CRP distribution in visit 1-4, by prognosis group. **(B)** Heatmap of the DMPs in the comparison between first and fourth visits (FDR < 0.05). Group, DAS28 and treatment are shown for every patient at the top, and the respective legend scales are shown to the right of the heatmap. Blue and red respectively indicate lower and higher methylation. Selected DMPs are highlighted at the right. **(C)** *FCGR* locus with PCHi-C interaction public data. (**D**) DNA methylation of cg11193201 and gene expression of *FCGR2A* in visits 1-4, by prognosis group. (**E**) *IL4R* locus with PCHi-C interaction data. (**F**) DNA methylation of cg02421308 and gene expression of *IL4R* in visits 1-4, by prognosis group. In (C) and (E), DMP and interacting HindIII fragments are shown below a genome browser annotation of transcripts and monocyte ChromHMM tracks (see Methods). DNA methylation and gene expression data from (D) and (F) were analyzed by bisulfite pyrosequencing and qRT-PCR, respectively. *RPL38* was used as the housekeeping gene (HKG). In (A), (D), and (F), each box represents the 25th to 75th percentiles. Lines inside the boxes represent the median. Lines outside the boxes represent the 25th percentile minus 1.5 times the IQR and the 75th percentile plus 1.5 times the IQR. In (D) and (F), pairwise group differences were evaluated by Wilcoxon tests. DAS28, disease activity score 28; DMARD, disease-modifying antirheumatic drug. HKG, housekeeping gene.

Supplementary Figure 3. DNA methylation changes and activity in blood and synovial monocytes in UA and RA. (A) Venn diagram showing overlap between DAS28-correlated CpGs and DMPs from Figure 2A. **(B)** Distribution of Spearman's correlation coefficient (ρ) in blood and SF monocytes ($p < 0.001$, $\rho \geq 0.7$). **(C)** Enrichment of DAS28-correlating CpGs in SF on DAS28-correlating CpGs in blood calculated with a Fisher's exact test. **(D)** Percentage of commonly correlated CpGs in blood and SF. **(E)** Selected examples of CpGs commonly correlated in blood and SF. Spearman's correlation coefficient (*ρ)* and p value are shown. **(F)** Sankey diagram showing

patient progression between the first and fourth visits. Colors indicate shifts in activity category between visits. The number of patients in every shift is indicated between brackets. **(G)** Schematic representations of the samples in the training and validation datasets used to derive the predictive regression model. **(H)** Violin plot showing the top 100 DAS28-correlated CpGs in UA and RA. Violin plots show density curves, and circles and vertical lines show the median and the 25th to 75th percentiles. The column facet indicates which data are contained in each violin. Left, top-correlated CpGs in UA; right, top-correlated CpGs in RA. The red background highlights the dataset in which the top-correlated CpGs have been selected. z-scored DNA methylation values of the other dataset are shown in an unsupervised fashion. Differences in the medians were verified by a Wilcoxon test.

Supplementary Figure 1

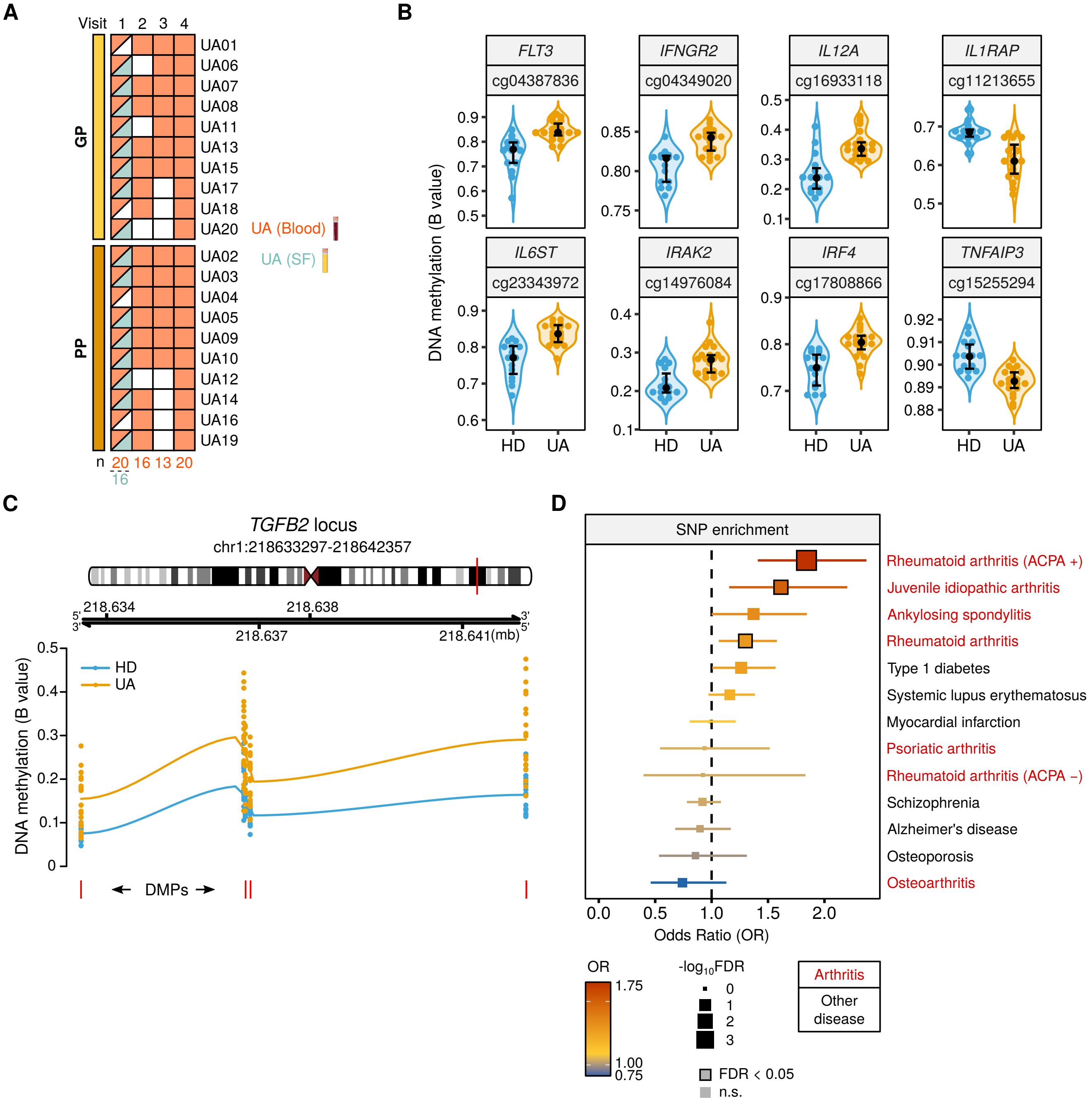

Supplementary Figure 2

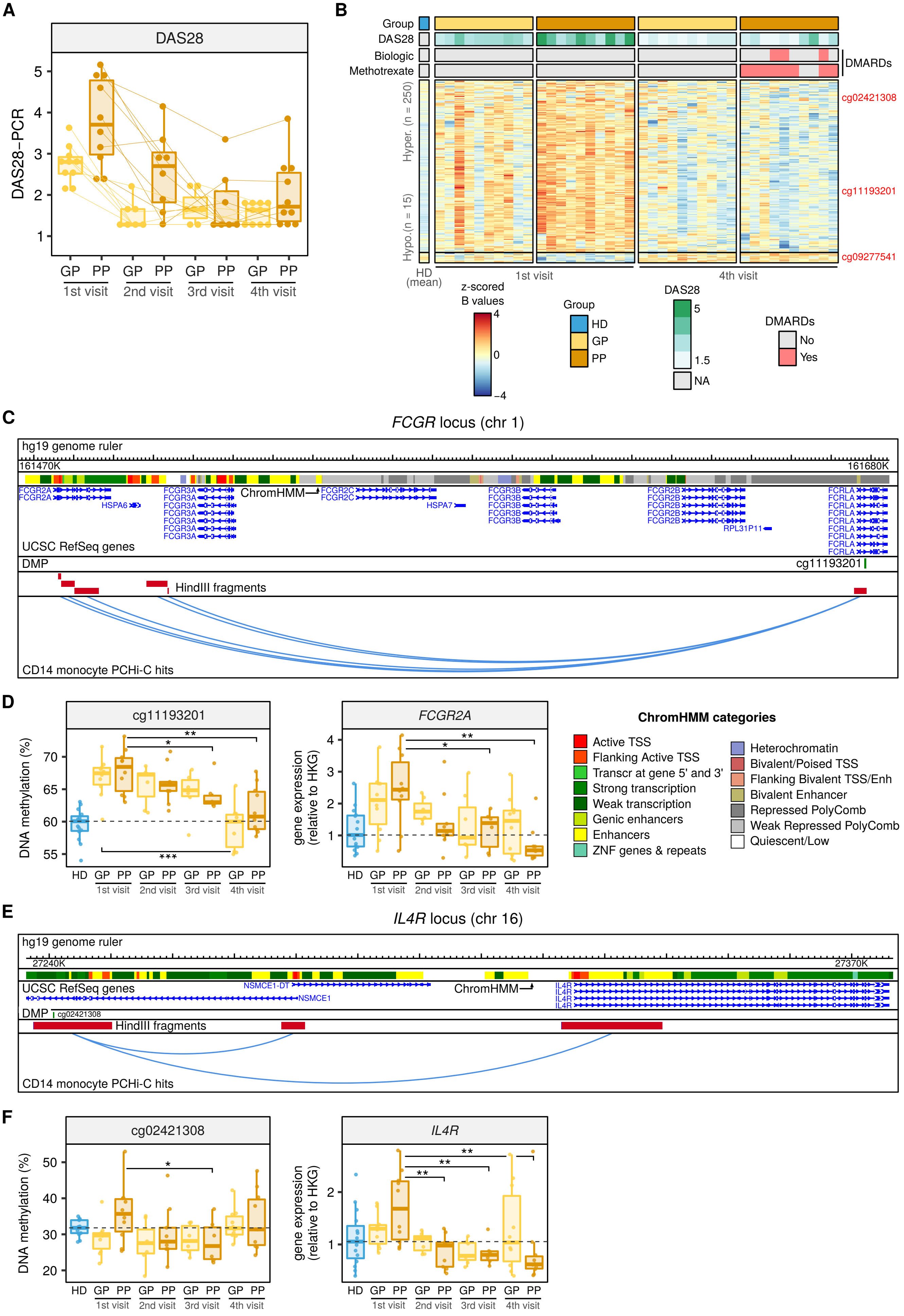

Supplementary Figure 3

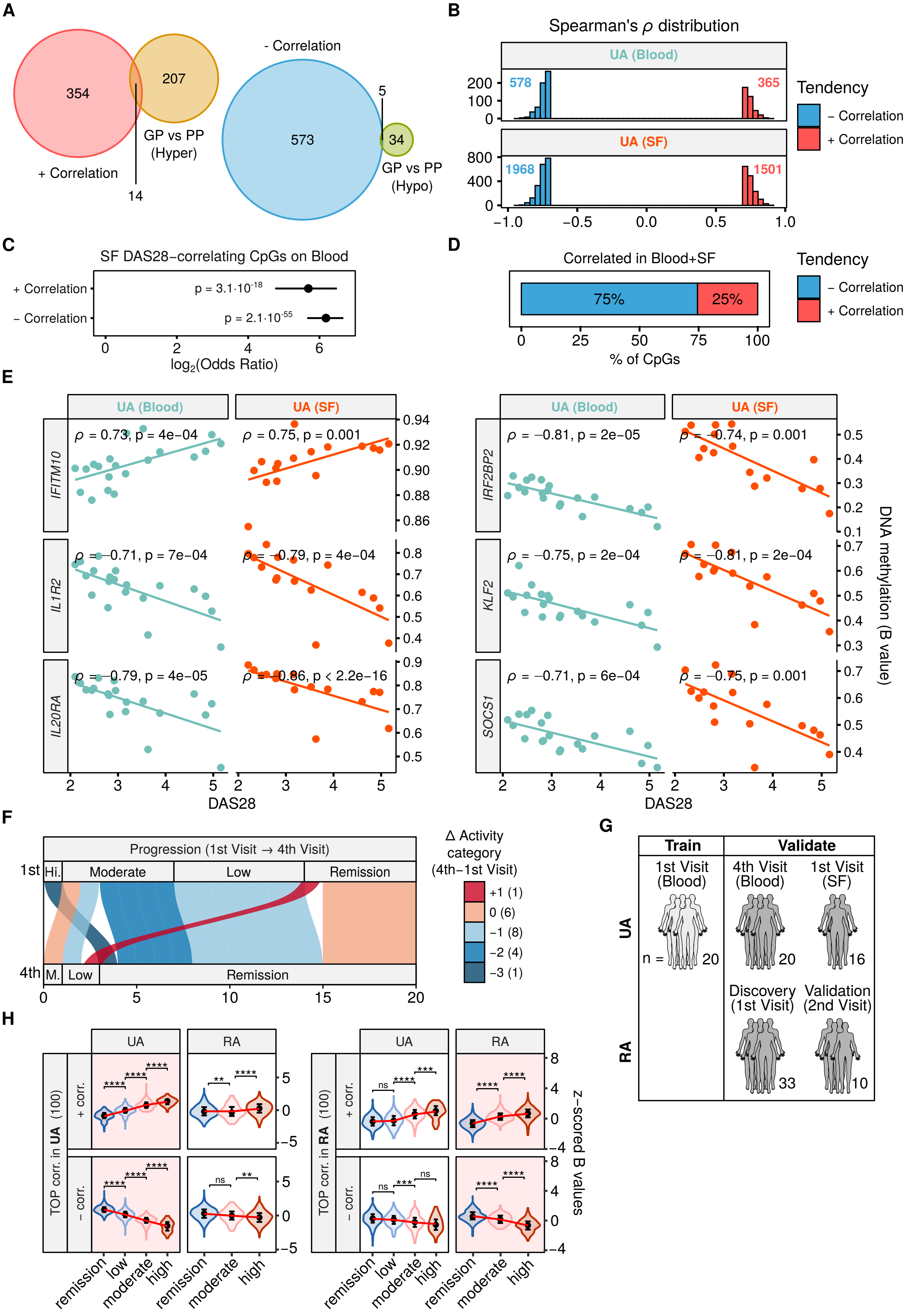

Supplement: Supplemental data [file jciinsight-7-158783-s190.pdf]
