# Supplementary material for: The synovial and blood monocyte DNA methylomes mirror prognosis, evolution, and treatment in early arthritis
Source: JCI Insight. 2022 May 9;7(9):e158783. doi: 10.1172/jci.insight.158783 (PMC9090240; doi:10.1172/jci.insight.158783)
Supplement: Supplemental table 1 [file jciinsight-7-158783-s191.pdf]

Supplementary Table 1

**A. HD vs. UA (Visit 1)**	level	HD	UA	p
n		15	20	
Age (mean (SD))		49.67 (15.40)	52.05 (13.80)	0.664
Sex (%)	M	5 (33.3)	8 (40.0)	0.960
	F	10 (66.7)	12 (60.0)	
**B. GP vs. PP (Visit 1, Blood)**	level	GP	PP	p
n		10	10	
Age (mean (SD))		52.00 (13.31)	52.10 (14.99)	1.000
Sex (%)	M	3 (30.0)	5 (50.0)	0.648
	F	7 (70.0)	5 (50.0)	
Duration (years, mean (SD))		0.54 (0.52)	0.35 (0.19)	0.307
DAS28 (mean (SD))		2.76 (0.45)	3.78 (1.06)	0.035
RF (%)	Negative	10 (100.0)	9 (90.0)	1.000
	Positive	0 (0.0)	1 (10.0)	
ACPA (%)	Negative	10 (100.0)	9 (90.0)	1.000
	Positive	0 (0.0)	1 (10.0)	
HLA - B27 (%)	Negative	6 (85.7)	7 (87.5)	1.000
	Positive	1 (14.3)	1 (12.5)	
**C. GP vs. PP (Visit 1, SF)**	level	GP	PP	p
n		8	8	
Age (mean (SD))		50.25 (13.48)	51.38 (15.86)	0.916
Sex (%)	M	5 (62.5)	4 (50.0)	1.000
	F	3 (37.5)	4 (50.0)	
Duration (years, mean (SD))		0.52 (0.59)	0.29 (0.13)	0.367
DAS28 (mean (SD))		2.82 (0.43)	4.06 (1.00)	0.021
RF (%)	Negative	8 (100.0)	8 (100.0)	NA
ACPA (%)	Negative	8 (100.0)	8 (100.0)	NA
HLA - B27 (%)	Negative	8 (100.0)	8 (100.0)	NA
**D. 1st vs 4th Visit (GP)**	level	Visit 1	Visit 4	p
n		10	10	
DAS28 (mean (SD))		2.76 (0.45)	1.56 (0.25)	<0.001
**E. 1st vs 4th Visit (PP)**	level	Visit 1	Visit 4	p
n		10	10	
DAS28 (mean (SD))		3.78 (1.06)	2.03 (0.84)	0.002

HD, healthy donor; UA, undifferentiated arthritis; GP, good prognosis; PP, poor prognosis; DAS28, disease activity score 28; RF, rheumatoid factor; ACPA, anti-citrullinated protein antibody; HLA, human leukocyte antigen.

P value is derived by Wilcoxon tests for numeric variables and by chi-squared tests for categorical variables.
